# Evaluation of satisfaction among relatives of mentally disabled patients who were users of a dental care protocol under general anaesthesia

**DOI:** 10.4317/medoral.17069

**Published:** 2011-07-15

**Authors:** Alfonso Escribano-Hernández, José M. García-Garraus, Ignacio Hernández-García

**Affiliations:** 1 Técnico de Salud. Primary Health Care Department-Salamanca (Castilla y León, Spain); 2 Resident doctor. Primary Health Care Department-Salamanca (Castilla y León, Spain); 3 Resident doctor. Primary Health Care Department-Salamanca (Castilla y León, Spain)

## Abstract

Objectives: In the field of action of disease associated with dependence the Third Health Plan of Castilla y León aims specifically at promoting the adjustment of health assistance to the needs of disabled people, according to their situation. Our objectives were: General: To know the satisfaction level among relatives or caregivers of people who were treated according to a protocol of dental care for mentally disabled people. Specific: To know if satisfaction is related to any sociodemographic characteristics of patients or to their pathology. Study design: Cross-sectional study by telephone survey, set in the Primary Health Area of Salamanca. The target population includes relatives or caregivers of mentally disabled patients who were sent to the hospital for treatment under general anaesthesia after being attended in Primary Dental Care Units, from 1st of June/2005 to 31st of May/2006. Social and demographic variables and patients’ diseases, as well as level of satisfaction with the service, were studied through a survey. Results: 67.4% of patients’ relatives or caregivers answered the survey, among whom 94.7% (C.I. 95%: 89-100%) were quite or very satisfied with the service in general. Conclusion:The protocol has high acceptance despite its difficulties and it has achieved considerable improvements in several aspects of patients’ life. This level of satisfaction was not related to any sociodemographic or clinical patient characteristics. Nevertheless, accessibility aspects and communication with patients may still be improved.

** Key words:** Health care surveys, dental care for disabled, patient satisfaction.

## Introduction

 Problems related to dental hygiene are the sixth most frequent condition which causes restrictions to mentally disabled people, and their prevalence is estimated to be 451/1000 in those patients (1). Caries, gingivitis and periodontal disease are the most outstanding oral health problems among mentally handicapped people (2).

Due to a lesser stress tolerance of these patients, some problems are almost invariably present in their dental management (3,4). Anyway, treatments could and should be carried out, if necessary, by adopting some special measures to control behavioural problems, such as general anaesthesia, deep intravenous sedation or ambulatory treatment with anxiolytic premedication (5).

Moreover, these people have worse oral health and higher proportion of untreated or poorly treated caries than the rest of the population, which suggests accessibility problems to dental services (6).

The Spanish Act 8/2003, of 8th of April, about citizen’s rights and duties related to health (Official Bulletin of the State n. 103 of 30th of April 2003), emphasizes the right of some groups of people to have access to special health programs, especially for disabled people.

Nevertheless, there are wide differences in dental care offered to disabled people in different regions (7).

In Castilla y León, in order to allow disabled people to receive the same public health service provision as other citizens, the Decree 142/2003 (Official Bulletin of Castilla y León nº 249 of 24th of December 2003) set out some measures to facilitate those services to those who need them. Disabled people who can not keep the necessary self-control to receive proper dental care will be sent to those health grounds where dental care provision can be guaranteed.

In the field of action of disease associated with dependence the Third Health Plan of Castilla y León aims specifically at promoting the adjustment of health assistance to the needs of disabled people, according to their situation.

For this reason, in Salamanca a protocol for which mentally disabled patients are seen in Primary Dental Care Units has been established. If according to primary odontologist opinion they need general anaesthesia for diagnosis or treatment, they are sent to the Area Hospital, where care is provided jointly by primary and secondary health care workers.

This protocol has already been evaluated from a quantitative point of view describing the pathology treated and suffered by patients (2). However, other aspects of medical care - such as satisfaction of patients or their relatives when those can not express their opinion - still remain to be studied.

The success of the protocol depends on its capability to fulfil the target population needs. However, satisfaction with health services is a complex issue related to a great variety of factors like lifestyle, previous experiences, future expectations and personal and social values (8).

Nevertheless, despite this complexity, Vuori (9) proposes some ethical considerations from the patient point of view which justify the inclusion of satisfaction in the quality evaluation.

Moreover, satisfaction analysis provides health workers and managers with information on those aspects of the organization that are perceived by population as unsatisfactory and that can be improved by modifying circumstances, behaviours or attitudes in the environment of the caregiving process (10).

The additional issue that disability is an independent risk factor for dissatisfaction with health care (11), specially in relation with accessibility, timetable flexibility and follow-up, must be taken into account. That is why efforts should be made to facilitate health care accessibility to disabled people and to evaluate its results in order to correct any deficiency.

This study precisely aims at knowing satisfaction level among relatives or caregivers of people who were treated with the protocol of dental care for mentally disabled people. As a secondary objective we want to know if satisfaction is related to any demographic variable or to the mental or dental pathology suffered by patients.

## Material and Methods

Study Design: Cross-sectional study by telephone survey.

Setting: Primary Health Care administration in Salamanca (Spain).

Participants: relatives or caregivers of mentally disabled patients who were sent to the hospital and treated under general anaesthesia after being attended in Primary Dental Care Units, from 1st of June/2005 to 31st of May/2006. In that period 108 patients were sent to the hospital because they met the requirements (general anaesthesia need for diagnosis or treatment). Only 86 of them were eventually treated under general anaesthesia. This population size in enough to estimate at least an 85% proportion of satisfied persons, with a standard error of 7.5% and a 95% confidence level.

**Figure 1:**
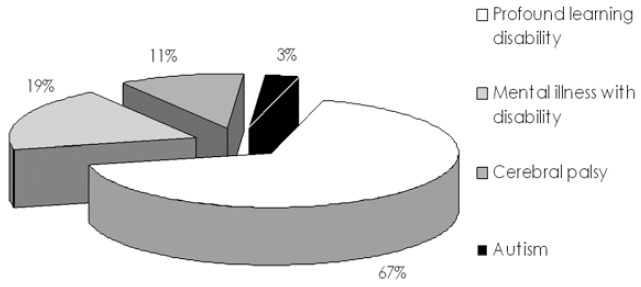
Patient’s pathologies.

Their characteristics have already been published (2). The distribution of their mental pathologies is shown in (Fig. 1).

Their mean age was 31 years old (standard deviation: 13.9), and 56% were male.

Independent variables: age, gender, mental disability of the patient, dental diagnosis and relationship between the patient and the person who answered the survey (relative, caregiver, etc.).

Dependent variables: result of the questions of a satisfaction survey fulfilled through bibliographic search in MEDLINE and with the subsequent work of the authors.

The following terms: “Disabled Persons”, “Mentally Disabled Persons”, “Dental Care for Disabled”, “Health Care Surveys”, “Dental Health Surveys”, “Questionnaires” and “Patient Satisfaction” were searched in MEDLINE, both in MESH and title and through different combination strategies.

The first 16 questions (that are included in the Results) use a Likert scale with the values: nothing, poor, fair, quite, a lot. It is possible to specify openly the changes perceived due to the intervention in question number 14. The last two are the following open questions:

What would you add to improve this service?

What would you remove to improve this service?

The survey was made between the 15th of January and the 15th of February/2008.

Questions were made by a trained health professional, using telephone numbers available in the clinical records of the patients treated through this protocol. We made a maximum of five attempts with each telephone number, waiting until seventh ringing tone.

Data were included in a Microsoft Access database, and were revised and statistically treated with SPSS.

Statistical analysis: we made a descriptive analysis of the dependent and independent variables. Quantitative data were analysed using measures of central tendency and statistical variability, and frequency distribution for categorical data.

The inferential statistics to find relations between satisfaction and the independent variables were made using ANOVA and chi-square tests, since satisfaction was considered a categorical variable with 5 categories.

It was also studied whether not finding the relatives or not answering the survey was in relation with any independent variable. In this case we used chi-square and Student’s t-tests.

## Results

The survey was answered by 58 patients’ relatives or caregivers (67,4% of total). We could not get data from the other 28 patients because we could not get in touch with them, it was never due to refusal to answer.

Among the 58 respondents, 68% were father or mother, 20% were brother or sister, 6% were brother or sister in law, 2% were wife and 4% institution assistants.

The results of the first 16 questions are shown in ( Table 1).

Besides, 30 respondents said that the patient showed changes as a result of the intervention different from those asked for in questions 9 to 13. These changes are shown in ( Table 2).

To the question “What would you add to improve this service?”, 8 persons answered:

Quicker care (3 persons)

Possibility of implants (1 person)

Annual examinations (3 persons)

To have different schedule than non disabled patients, who sometimes got annoyed (1 person)

And to the open question “What would you remove to improve this service?”, only 2 people answered, one in relation with the need of going to the hospital and the other with the shortage of staff.

94.7% (95% confidence interval: 89-100%) of polled people were quite or a lot satisfied with the service in general.

That general satisfaction with the service was not statistically related with any demographic variable (age or gender), neither was it related with the pathologies suffered by patients (p>0.05).

The impossibility of contact with a relative or caregiver was not related with any of those independent variables (p>0.05).

## Discussion

We made the satisfaction survey without outstanding problems and obtained the opinion of 67.4% of relatives or caregivers polled. We think it is an acceptable figure given the data in other studies (12,13), although there are others with a better response rates (14,15).

We think that the approach to get directly the opinion of relatives or caregivers of disabled people is an added value.

Besides, characteristics of not surveyed persons were not different from those who did answer.

**Table 1:**
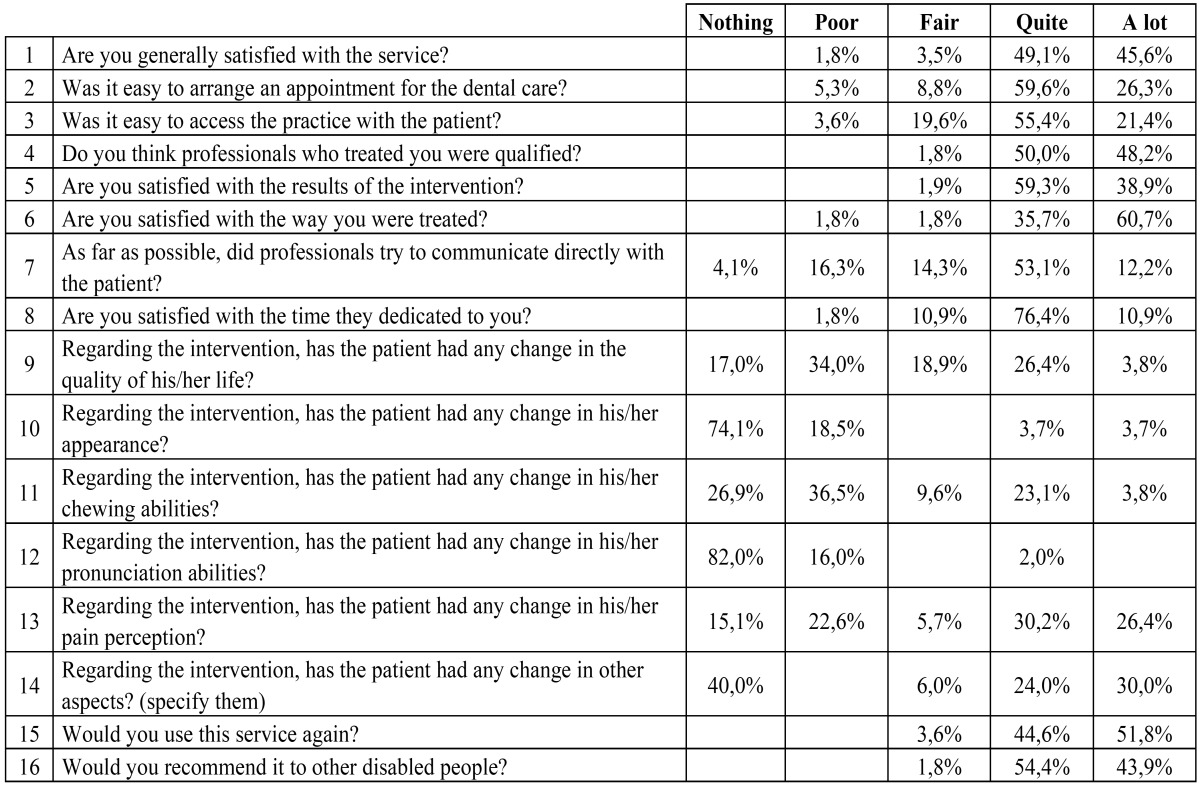
Results of the first 16 survey questions (N: 58).

**Table 2:**
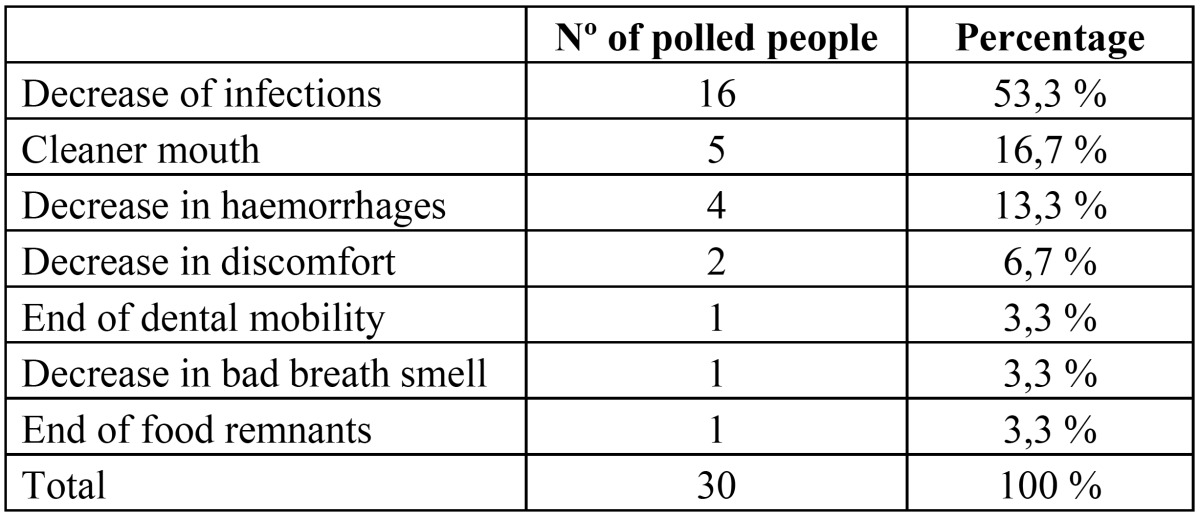
Nº of polled with changes in other specific aspects (survey question number 14).

The great majority (90%) were first or second degree relatives of patients.

Satisfaction with the service as a whole was very high - over 89% - taking into account that it is a newly established service. Other studies have also found high levels of satisfaction (higher than 85%) after the treatment of disabled patients (14).

Nevertheless, we have seen that interventions achieved little functional changes in many cases (63.4% of cases in the chewing abilities and 98% of cases in pronunciation) and that the cases which declared quite or a lot of change were very little in pronunciation (2%) and some more in the chewing improvement (26.9%).

Becker et al. (14) also studied the impact of interventions in functional changes. They were remarkable only in 15% of cases and there were no changes at all in 25% of them.

Besides, we found scarce changes in the physical appearance (74.1% had no change at all and only 7.4% had quite or a lot of changes). However Becker et al. (14) noted positive change in the facial appearance in 63% of cases.

Anyways, despite these little functional and aesthetic changes that all of us have found, not only the general satisfaction is high but also both Becker et al. (14) and us have seen that relatives and caregivers would use the service again and they would recommend it to other patients.

This apparent paradox between little functional changes and high general satisfaction might be explained with the shortage of services specifically directed to these patients helps the bias towards a high level of satisfaction when a service comes to fill this gap (8,11). And these patients’ relatives are likely not to have an “ideal” objective but a moderate one (16), to improve functionality, aesthetic aspect and social acceptance. We think that the moral and ethical justification for this kind of interventions is evident, as other authors do (16,17).

We also investigated about pain perception and quality of life. And they had improved between fair and a lot in 49% and 62.3% of cases respectively. We have found no bibliographic references to compare with, but we think these two questions may have influence in general satisfaction.

When analysing other questions of the survey, we can appreciate that there are some aspects in the setting of the protocol that are worse appreciated and therefore can be improved.

Particularly, about accessibility to the service there was 14.1% and 23.2% of poor-fair satisfied with the ease of getting an appointment and with the ease of access at the practice with the patient, respectively.

Regarding dedicated time, 12.7% of polled were poor-fair satisfied.

However, accessibility problems are also described by other authors (6,11).

An aspect we specifically wanted to study was if health professionals communicated directly with the patient when possible. To that question, 31% of surveyed persons answered poor or fair and even a 4% answered that nothing. Other studies have shown that communication problems are the most important barrier to the treatment of these patients (18,19). Besides, Jongh et al. (18) found that these problems were particularly true for ethnic minority groups. We have not studied the ethnic characteristic because there was no variability in our sample population. Nevertheless we studied if there was any difference in satisfaction in relation with other variables such as age, gender, mental disability of the patient and dental diagnosis and we could not find any significant difference. We have not found bibliographic references which analyse differences in satisfaction in relation with those variables.

Inherent difficulties to communication with these patients are obvious. Nevertheless we think it is important to make an effort to improve communication with them, thus achieving a higher quality of care.

Furthermore some people expressed changes in other relevant aspects: 16 had a decrease in the number of infections, 7 patients had a cleaner mouth with better breath smell and 4 mentioned a decrease in haemorrhages. Other studies evaluating dental care programs for disabled people also show similar results as decrease in stomatitis (21) or in the number of bleeding sites (22).

The two final open questions of this study did not provide much information.

In short, we could get the opinion of a high percentage of relatives or caregivers among those we tried to survey.

We can conclude that the protocol has a high acceptance in general despite its difficulties and it has achieved valuable improvements in many aspects of these patients’ life, not related to age, gender, intervention or pathologies suffered by them.

However we can still improve some aspects like accessibility and communication skills. 
